# Psychometric Properties of the Dutch Child Avoidance Measure

**DOI:** 10.1007/s10578-023-01517-w

**Published:** 2023-03-13

**Authors:** Ellin Simon, Henriëtta J. Bragt-de Jong, Petra Butler, Stephen P. H. Whiteside

**Affiliations:** 1 Department Clinical Psychology, Open University, Heerlen, The Netherlands; 2 Department of Medical and Clinical Psychology, Tilburg University, Tilburg, The Netherlands; 3 Department of Psychiatry and Psychology, Mayo Clinic, Rochester, USA

**Keywords:** Avoidance, Anxiety, Child, Child Avoidance Measure, Psychometric properties

## Abstract

Avoidance is considered a hallmark feature of child anxiety, but convenient measures are scarce. This study examined the psychometric properties of the Child Avoidance Measure (CAM) in a Dutch population, focusing mainly on the child-version. We included children 8 to 13 years old from a community sample (*n* = 63, longitudinal design) and a sample of high-anxious children (*n* = 92, cross-sectional design). Regarding the child-version, the internal consistencies were acceptable to good with moderate test-retest reliability. The validity analyses showed encouraging results. High-anxious children had higher avoidance scores than children from a community sample. Regarding the parent-version, both the internal consistency and test-retest validity were excellent. Overall, this study confirmed the sound psychometric properties and usefulness of the CAM. Future studies should focus on the psychometric properties of the Dutch CAM in a clinical sample, assess its ecological validity more extensively, and examine more psychometric features of the parent-version.

## Introduction

Avoidance has always been necessary for survival (e.g., [1]), encompassing ‘any act or series of actions that enables an individual to avoid or anticipate unpleasant or painful situations, stimuli or events, including conditioned aversive stimuli’ [2]. Avoidance becomes problematic if the avoided stimuli are benign [3], or when avoidance become excessive, interfering with daily-life functioning [4, 5]. Avoidance plays a central role in the development (e.g., [6]), maintenance (e.g., [7]) and return of fear [3], and is considered a hallmark feature of specific fears, broader trait anxiety [8] and anxiety-related difficulties [3].

Anxiety is a normal emotional response that signals threat. Anxiety can take many forms, such as worrying about certain topics (e.g., global warming), fearful reactions in response to specific feared stimuli (e.g., spiders). Anxiety can be adaptive for a certain developmental phase or given a certain situation, such as an objective trauma. However, disproportional or enduring anxiety can lead to anxiety-related difficulties or even to anxiety disorders (e.g., [9]). Moreover, anxiety is a central emotion in anxiety-related disorders such as post-traumatic stress disorder (PTSD).

In the short-term, avoidance reduces feelings of anxiety. However, avoidance prevents children from learning that the anticipated threat does not actually occur, thus negatively reinforcing and maintaining the fear in the long run [5, 10]. For example, children with a fear of dogs might evade an approaching dog, thus avoiding the perceived threat, alleviating the anxiety in the short-term but sustaining the anxiety in the long-term (i.e., not learning that most dogs are benign and thus staying afraid of dogs). Therefore, in the long run, children most likely remain anxious when responding with avoidance [11]. Additionally, avoidance can hinder adequate emotional processing and learning [12]. Moreover, a recent systematic review showed that enduring avoidance is predictive of the chronicity of anxiety disorders [13]. During exposure-based interventions and treatments, children are encouraged to reduce their avoidance. However, after exposure-based interventions or treatment, returning to avoidance may reestablish fear [3, 14, 15].

Avoidance is a transdiagnostic factor and active therapeutic target for problems with anxiety, such as anxiety-related disorders and PTSD [4], where the expression of avoidance can either be behavioral, experiential, or both. Behavioral avoidance has a predominantly overt character and occurs when children physically avoid certain situations that increase their anxiety, such as sleeping over at a friend’s house. Experiential avoidance is more covert in nature and occurs when children avoid or adapt unwanted personal experiences (e.g., painful memories), and contexts associated with these unwanted experiences [16].

Despite the relevance of avoidance in the etiology and continuity of anxiety-related difficulties, convenient measures to assess avoidance in anxious children are scarce. Observational measures, such as Behavioral Avoidance Tests [17] are prone to biases due to demand characteristics and social desirability, show difficulties with standardization, and are time consuming [18]. Motion-tracking measures, like the Yale Interactive Kinect Environment Software platform (YIKES, 18), may not possess good cost-to-benefit ratios. Structured interviews, such as the Diagnostic Interview for Mental Disorders in Children and Adolescents (Kinder-DIPS, 19), are time consuming and need trained interviewers. In contrast, questionnaires are convenient, time-efficient measures that possess a good cost-to-benefit ratio.

A number of questionnaires exist to measure avoidance limited to a specific type of anxiety. For example, the Severity Measure for Specific Phobia assesses avoidance related to specific phobia [20], the Social Anxiety Scale for Children-Revised (SASC-R, 21) and the Social Anxiety Scale for Adolescents (SAS-A, 22) assess avoidance related to social anxiety, and the Screen for Child Anxiety related Emotional Disorders (SCARED, 26, 27) assesses school avoidance. More broadly, the Mutidimensional Anxiety Scale for Children (MASC, 23) and MASC-2 [24] measure harm avoidance [25]. A different type of questionnaire on anxiety related avoidance is the Anxiety and Avoidance Scale for Children (AVAC, 28). The AVAC includes only individualized items, making it highly suitable to monitor individual changes in avoidance. The child and parent are asked to report three of the child’s most anxiety provoking situations, to specify the child’s avoidance behavior in each of these situations, and to rate the severity of the child’s anxiety and avoidance in these three situations. Although valuable, none of these questionnaires can be applied to all types of anxiety related avoidance and are suitable to examine individual as well as interindividual changes in avoidance.

In contrast, the Child Avoidance Measure (CAM, 29) is a questionnaire that assesses the level of avoidance related to all types of anxiety and can be used to measure individual and interindividual changes in avoidance. It measures active avoidance, passive avoidance (i.e., overt and covert) and refusal. The questionnaire can be completed by the child, i.e., the Child Avoidance Measure-Self Report (CAMS), as well as by the parent, i.e., the Child Avoidance Measure-Parent Report (CAMP). The original CAMS and CAMP possess good internal and test-retest reliability, concurrent and criterion validity, and treatment sensitivity [29]. In the current study, we sought to examine the psychometric properties of the Dutch version of the CAMS questionnaires. We expected to confirm the sound psychometric properties of the CAMS using a Dutch version in a Dutch population.

## Method

### Participants

To examine avoidance in children 8 to 13 year old with varying levels of anxiety, we recruited two separate samples of Dutch speaking children: one sample from the general population (i.e., community sample) and one sample from the high-anxious population (high-anxious sample). An a priori power analyses with G*Power 3.1.9.7 [30] showed that for correlation analyses and t-tests, with *α* = 0.05, two-tails, *r* = .4 and a power of 0.90, we needed to include 61 children in both samples. Base on this criteria, the obtained sample sizes had adequate power, with *n* = 63 and *n* = 92 for the community and high-anxious samples, respectively. The descriptive statistics of both samples are presented in the results section.

#### Community Sample

The recruitment of the community sample (*n* = 63) took place at various locations in the Netherlands via convenience sampling in the personal networks of participating psychology students. That is, the parents of participating children, as well as children aged 12 years and older, signed active informed consent forms, which they handed to the psychology master student. Except for the age and language criteria, there were no exclusion or inclusion criteria.

#### High-anxious Sample

The recruitment of the high-anxious sample (*n* = 92) took place at various locations in the Netherlands via press releases, interviews, social media, and via flyers. In order to take part, parents as well as children aged 12 years and older had to sign informed consent forms. Thereafter, a research assistant provided an account to the participants that gave them full access to an online platform. We then followed a selection procedure consisting of a screening questionnaire and a diagnostic interview to identify high-anxious children. In addition to the age and language criteria, there were two inclusion criteria: (1) children reported high anxiety symptomatology on the screening questionnaire (boys > 27; girls > 36); (2) children did not have significantly invalidating, but only mild to moderately severe anxiety diagnoses on the diagnostic interview. Children who did not complete the diagnostic interview as well as the CAMS, were excluded from data analyses.

### Procedure

The study in the community sample had a longitudinal design. The children completed the CAMS, the 8-item Avoidance Fusion Questionnaire for Youth [31], and the Youth Anxiety Measure for DSM-5 [32] at baseline, as well as at day 30, which took them approximately 20 min on both occasions. At the same days, the parents completed the parent report version of the avoidance questionnaire, the CAMP. Additionally, the children kept an avoidance diary over the 30 day period, which took them 1 min per day. The participants sent the anonymized questionnaires and avoidance diaries directly to the principal investigator (first author) by using stamped envelopes. Consistent with ethical standards, children could end their participation at any point without having to provide a reason. The study was conducted under full IRB approval from the local ethical committee, U2019/02802/MQF.

The study in the high-anxious population had a cross-sectional design and was part of an overarching study [11], which was preregistered at https://osf.io/g2avh. Avoidance was measured via the CAMS and the diagnostic interview. The diagnostic interview was provided by a trained interviewer via telephone. The trained interviewers were required to hold a bachelor degree in Psychology. Thereafter, children completed the CAMS on an online platform https://leertedurven.ou.nl. The online platform ran under the https protocol for security reasons. Privacy was GDPR-compliant. Secure information transfer was handled by using a separate server for the user information database. We obtained ethical approval for this study by a national medical ethical review board in the Netherlands, NL60801.068.17. Table 1 shows the psychometric properties that were assessed within each sample.

**Table 1:** Assessed Psychometric Aspects of the Child Avoidance Measure Self-report (CAMS) for Community and High-anxious Sample

	Community sample *N* = 63	High-anxious sample *N* = 92
Internal consistency^1^	X	X
Test-retest reliability^2^	X	
Child-parent correspondence^3^	X	
Convergent validity I ^4^	X	
Convergent validity II^5^		X
Ecological validity^6^	X	
Discriminant validity^7^	X	X

Note: ^1^assessed with CAMS at baseline; ^2^assessed by comparing the CAMS at baseline to CAMS at day 30; ^3^assessed by comparing CAMS to Child Avoidance Measure Parent-report (CAMP); ^4^asssessed by comparing CAMS to 8-item Avoidance Fusion Questionnaire for Youth (AFQ-Y) and to the avoidance score on the Structured Child ; ^5^assessed by comparing CAMS to avoidance in Structured clinical interview for DSM-5 disorders for children (SCID-5-Junior); ^6^assessed by comparing CAMS to avoidance diary; ^7^assessed by comparing the CAMS scores between the community sample and the high-anxious sample.

### Measures

#### Avoidance

*The Child Avoidance Measure Self report (CAMS) and Parent report (CAMP)* assess avoidance related to anxiety, fear or worry in children [29]. The CAMS concerns the child self-report version, whereas in the CAMP, the parent reports about the child’s avoidance. The development of the original CAMS and CAMP [29] began with rational item generation during which two psychologists with expertise in child anxiety disorders created a 14-item pool based on an operational definition of the target construct. This item pool was reduced to eight items based on examination of correlations, item performance, and exploratory factory analysis within a sample of 106 children and their parents. Subsequently, the reliability and validity of the resulting items were examined with community and clinical samples [29].

The original CAMS and CAMP consist of eight items that are preceded by a general stem statement: “When I/my child feel(s) scared or worried about something …”. The items measure passive avoidance (e.g., “…I/he or she try/tries not to go near it/avoid it”), active avoidance (e.g., “…I feel scared until I get away from it”/”…He or she tries to get away from it”), and refusal (e.g., “…I refuse to do it”/”… he or she asks me to take care of it for him/her”), The items are scored on a 4-point scale (0 = “almost never” to 3 = “almost always”), with scores ranging between 0 and 24. We translated the CAMS and the CAMP into Dutch via a translate and back-translation procedure.

The original CAMS and CAMP possess good reliability and good internal consistency, concurrent validity (both with a pre-existing measure of avoidance and in differentiating between clinical and community samples), criterion validity, and treatment sensitivity [29]. The CAMP especially possesses good predictive validity of worsening anxiety over time. The reliability and validity of the Dutch version of the CAMS, and the reliability of the CAMP are reported in the current study.

*Avoidance and Fusion Questionnaire for Youth (AFQ-Y8*, 33): We used the 8-item version of the AFQ (i.e., AFQ-Y8) for youth to get insight into experiential avoidance in children (regardless of the presence of anxiety). The AFQ-Y8 was translated in Dutch and a psychometric study [34] confirmed the usefulness of applying the AFQ-Y8 in the current age group. We used the child self-report, which consists of 8 items (e.g., “I don’t try out new things if I’m afraid of messing up”). These items could be answered on a 5-point Likert scale ranging from 0 (not at all true) to 4 (very true). The possible range of scores is 0–32 (min-max). The Dutch version of the AFQ-Y8 has good psychometric properties [34]. In the current study, the internal consistency of the child self-report was α = 0.61, and the internal consistency of the parent report was α = 0.85. The AFQ-Y8 was used only in the community sample.

##### Avoidance Diary

During the 30-day period, children completed a self-report diary on their avoidance. Every day, the child reported if (s)he avoided something or not that specific day (yes/no). The avoidance diary was used only in the community sample.

##### Structured Clinical Interview for DSM-5 Disorders for Children (SCID-5-Junior)

The SCID-5-Junior [35] is a structured clinical interview to measure the most common DSM-5 disorders in children, including the anxiety disorders. Consistent with the DSM-5 [4], avoidance (e.g., “Do you try to avoid certain things, so that you will not get a panic attack?”) is an obligatory criterion for every anxiety disorder except for generalized anxiety disorder. The minimum amount of avoidance criteria that could be obtained is 0, the maximum amount cannot be specified as, in theory, children could have an unlimited number of specific phobias (see ‘Descriptive statistics’ for the range in the current study). By adding the number of avoidance criteria we obtained a continuous measure of avoidance, with higher scores reflecting more avoidance. The SCID-Junior is a DSM-5 update of the DSM-IV-based Kid-SCID [35–37] which has been widely applied to assess childhood mental disorders, and has moderate to good interrater reliability and internal consistency. The SCID-5-Junior was used only in the sample of high-anxious children.

#### Anxiety

##### Youth Anxiety Measure for DSM-5 (YAM-5)

We assessed child anxiety symptoms with the Youth Anxiety Measure for DSM-5 (YAM-5, 32, 38). This questionnaire is suitable for children aged 8–18 years. We used the child self-report, which comprises two parts. The first part (28 items) taps all major DSM-5 anxiety disorders (e.g., I am afraid to go anywhere without my parents) and the second part (22 items) taps specific phobias (e.g., I am afraid of people who are dressed up in costumes). The items are rated on a 4-point scale that ranges from 0 (never) to 3 (always). Earlier studies (e.g., [32, 38]) reported good internal consistencies, good test-retest reliability, good concurrent validity and good construct validity. In the current study, the internal consistencies for the child and parent report was α = 0.88. In the current study, the YAM-5 was used only in the community sample.

### Analyses

With respect to data preparation, no missing data needed to be imputed in the high-anxious sample, as items were all mandatory in the online platform. For missing data resulting from the pen-and-paper method in the community sample, we imputed missing data as follows. Missing items were imputed with the item’s median if the child had only one missing item on the CAMS, CAMP, or AFQ-Y or maximally five missing items on the YAM-5 questionnaire. There were no children with more missing items. In addition, if variables were not normally distributed, we first applied nonparametric alternatives, and then normalized the non-normal variables (reduction to *M* +/- 2 *SD*) and ran the analyses again. Outcomes of non-normalized data were primarily reported, followed by a description of different outcomes with normalized data.

The following analyses were performed. First, the internal consistencies of the CAMS and the CAMP were determined with Cronbach’s alpha as well as with the intra-class correlation (ICC) based on a one-way random-effects model. Second, the test-retest reliability of the CAMS and CAMP were calculated with the ICC based on a two-way mixed-effects model. Third, ICC’s were calculated to gain insight in the child-parent correspondence of the CAMS based on a one-way random-effects model. Fourth, the convergent validity of the CAMS was calculated with Pearson r correlations. Fifth, the ecological validity of the CAMS was examined with Spearman’s rank correlations. Finally, the comparison between the children from the community and the high-anxious sample on the CAMS was calculated with an independent sample t-test.

Cronbach’s alpha was interpreted as follows: < 0.50: unacceptable; > 0.50 poor; > 0.60: questionable; > 0.70: acceptable; > 0.80: good, and > 0.90: excellent [39]. The ICC’s were interpreted as: < 0.50: poor; > 0.50: moderate; > 0.75: good; > 0.90: excellent [40]. Correlations were interpreted as follows: in the order of 0.10: small; in the order of 0.30: medium; in the order of 0.50: large [41]. Cohen’s *d* [42] was calculated as effect size estimate for the independent t-test, which was interpreted following Cohen’s rule of thumb: small (*d* = 0.20), medium (*d* = 0.50) and large (*d* = 0.80). We used IBM SPSS Statistics for Windows, version 26 (IBM Corp., Armonk, N.Y., USA) and applied a significance level of *p* < .05 for all analyses.

## Results

### Descriptive Statistics

The community sample (*n* = 63) consisted of 41 girls (65%) and 22 boys (35%), with a mean age of 10.38 (*SD* = 1.57). The high-anxious sample (*n* = 92) consisted of 50 girls (54%) and 42 boys (46%), with a mean age of 9.77 (*SD* = 1.55). Both samples included children from various parts of the Netherlands. We checked all of the data for possible gender and age effects. Girls (*M* = 36.31, *SD* = 16.54) had significantly higher self-report scores on the YAM-5 at baseline than boys (*M* = 22.24, *SD* = 12.84), *t*(58) = -3.143, *p* = .003, and after 30 days (Girls: *M* = 31.44, *SD* = 15.83; Boys: *M* = 18.89, *SD* = 16.56), *t*(48) = -2.58, *p* = .013. In addition, parents (CAMP) rated the avoidance scores at baseline higher for girls (*M* = 13.05, *SD* = 4.68) than for boys (*M* = 10.18, *SD* = 3.90), *t*(61) = -2.10, *p* = .040. No other gender effects in either sample were found, all *p*’s > 0.10, nor any age effects, all *p*’s > 0.05. We also checked the normality of the data. All variables had a distribution that approximated normality, except for daily avoidance with skewness = 2.043, (SE = 0.304), and kurtosis = 4.66 (SE = 0.599). Table 2 shows the descriptive statistics and correlations between variables.

**Table 2:** Associations between the included constructs at pre- and posttest in the Community Sample

Community sample (*n* = 63)									
	CAMS base	CAMP base	AFQ-Y base	YAM-5 base	AD	CAMS post	CAMP post	AFQ-Y post	YAM-5 post
CAMS^a^ base^b^	-								
CAMP^c^ base	0.38**	-							
AFQ-Y^d^ base	0.37**	0.26*	-						
YAM-5^e^ base	0.22	0.34**	0.57**	-					
AD^f^	0.20	0.09	0.07	0.07	-				
CAMS post^g^	0.59**	0.32*	0.27*	− 0.02	0.39**	-			
CAMP post	0.36**	0.54**	0.13	0.15	0.09	0.44**	-		
AFQ-Y post	0.36**	0.21	0.51**	0.21	0.24	0.32*	0.09	-	
YAM-5 post	0.26*	0.28*	0.51**	0.79**	0.11	0.43**	0.25	0.36*	-

Note: ^a^CAMS: Child Avoidance Measure Self-Report; ^b^Base: baseline; ^c^CAMP: Child Avoidance Measure Parent-Report; ^d^AFQ-Y: Avoidance and Fusion Questionnaire for Youth; ^e^YAM-5: Youth Anxiety Measure for DSM-5; ^f^AD: Anxiety Diary; ^g^Post: measurement after 30 days.

### Reliability

In the community sample, the internal consistencies of the CAMS were ⍺ = 0.75 and ICC = 0.74, 95% CI [0.62, 0.83], *p* < .001 at baseline, and ⍺ = 0.84 and ICC = 0.83, 95% CI [0.75, 0.89], *p* = .000 after 30 days. The internal consistencies of the CAMP were ⍺ = 0.90 and ICC = 0.89, 95% CI [0.84, 0.93], *p* = .000 at baseline, and ⍺ = 0.90 and ICC = 0.90, 95% CI [0.86, 0.93], *p* = .000 after 30 days. The test-retest reliability of the CAMS was ICC = 0.75, 95% CI [0.64, 0.84], *p* < .001. The test-retest reliability of the CAMP was ICC = 0.90, 95% CI [0.85, 0.93], *p* = .000. In the high-anxious sample, the internal consistencies of the CAMS were ⍺ = 0.75, and ICC = 0.71, 95% CI [0.61, 0.79], *p* = .000.

### Validity

The correlation between child and parent report was ICC = 0.26, 95% CI [0.17, 0.38], *p* < .001, at baseline. After 30 days, the child-parent correspondence was ICC = 0.37, 95 CI [0.27, 0.49], *p* = .000.

With respect to the convergent validity in the community sample, results showed significant positive correlations between CAMS and AFQ-Y8 scores both at baseline *r* = .37, *p* = .005, and after 30 days, *r* = .32, *p* = .015. Regarding the convergent validity in the high-anxious sample, we found no association between the CAMS scores and the avoidance score of the SCID-5-Junior, *r* = .17, *p* = .113.

Regarding the ecological validity there were no significant correlations between the baseline CAMS or CAMP scores and daily avoidance, both *p*’s > 0.10. However, CAMS scores at the end of the 30-day period had a significant positive correlation with daily avoidance, *r* = .39, *p* = .003.

Finally, regarding testing the discriminant validity between the community and high-anxious sample, it appeared that the community sample (*M* = 11.68, *SD* = 4.75) had significantly lower avoidance scores than the high-anxious sample (*M* = 14.04, *SD* = 4.71), *t*(174 ) = 3.18, *p* = .002; *d =* 0.50. Findings were not different after normalizing scores.

## Discussion

Avoidance is key in the development, maintenance and renewal of fear, and is considered a hallmark feature in child anxiety. Various interventions and treatments for childhood anxiety aim at reducing avoidance (e.g., exposure). Of the available Dutch language questionnaires to measure specific types of anxiety related avoidance (e.g., [21-24, 26, 27]), none are designed to measure avoidance across all types of anxiety presentations. Therefore, this study examined the psychometric properties of the Dutch version of the Child Avoidance Measure Self-report (CAMS, 19) in community and high-anxiety children in the Netherlands.

Overall, we confirmed the sound psychometric properties of the original CAMS/CAMP [29], using the Dutch translation. With respect to the reliability, the internal consistencies of the child-version of the CAM (CAMS) ranged from acceptable to good and the test-retest reliability was good. The internal consistencies of the parent-version (CAMP) ranged from good to excellent and the test-retest reliability was excellent.

Regarding the validity of the child-version of the CAM, results were mixed but encouraging. We found a small association between the children and parents as informants. Although this may seem weaker than one might expect, it is in line with other studies investigating child-parent agreement in reporting emotional and behavioural problems [43]. Generally, the more observable a behaviour is, the higher the agreement between self- and proxy reports. Possibly, due to the more covert avoidance items in the CAM, the agreement between the informants may be weaker. As such, it is of importance to include child-reports in less observable child problems, such as avoidance.

The assessment of the convergent validity revealed a significant moderate positive relation between the CAMS and the AFQ-Y8. Though generally speaking the cut-off showing adequate convergent validity is a strong correlation (i.e., > 0.50) [44], we consider the current correlations (0.37 and 0.32 on baseline and post-test, respectively) as encouraging results for supporting validity, as the AFQ-Y8 measures only experiential avoidance whereas the CAMS measures a more broadly defined construct of avoidance, including not only experiential but also behavioural avoidance. However, the avoidance levels assessed with the CAMS did not converge with the avoidance levels assessed with the diagnostic interview SCID-5-Junior. This is most likely due to the fact that the CAMS assesses overall, daily life avoidance related to anxiety, whereas the diagnostic tool examines avoidance only after the child reports severe anxiety. Additionally, the CAMS examines the level of avoidance, whereas the diagnostic tool only assesses whether avoidance is present (yes/no). This may indicate that the avoidance levels of the SCID-5-Junior reflect the diversity of anxiety problems (i.e., being anxious about and avoiding a variety of entities), rather than the depth of the avoidance symptoms.

With respect to the ecological validity, the baseline CAMS was not related to daily avoidance. Though, after completing the daily avoidance diary for 30 days, the CAMS had a moderate positive relation with daily avoidance. Similarly, the baseline CAMS was not related to child anxiety, whereas we found a moderate positive relation between the CAMS and child anxiety after filling in the daily avoidance diary for 30 days. Interestingly, whereas the CAMS avoidance levels within the community sample were not related to baseline scores of daily avoidance or child anxiety, there were positive associations between the CAMS avoidance levels on the one hand and daily avoidance and child anxiety on the other hand after 30 days. We want to tentatively suggest that an increased awareness and understanding of the concept of avoidance over the 30 days may explain the presence of a positive associations after 30 days, while these variables were unrelated at baseline. That is, filling in the avoidance diaries for 30 days may have increased the children’s awareness and understanding of the concept of avoidance. Measurement reactivity is particularly common in diary studies [45], and response shifts towards descriptions that are more realistic or accurate have been reported in diary studies (e.g., [46]).

Finally, the community sample had lower levels of avoidance than the high-anxious sample. The instrument thus seems to discriminate the levels of avoidance in children who exhibit anxiety problems and those who do not. This finding supports the CAMS’ discriminant validity.

### Theoretical and Clinical Implications

This results of this study support the validity of using the CAMS questionnaire in community samples as well as high-anxious samples. Also, we demonstrated the usefulness of the Dutch version of the CAMS. An important theoretical and clinical observation we made, was the poor agreement between levels of avoidance measured with the avoidance questionnaire and the diagnostic tool. If clinicians want to gain insight into children’s avoidance levels, using an avoidance questionnaire in addition to a diagnostic interview seems to be of added value. Future studies are needed to gain more insight into the unique information resulting from using questionnaires or diagnostic interviews.

### Limitations

The findings of this study should be interpreted in light of its limitations. First of all, we did not include a sample of clinically anxious children, which limits the generalizability of the findings. Nevertheless, we did include a sample of children from the general population, as well as a sample of children who were high-anxious. In both samples, the psychometric properties are promising, which is encouraging. Second, the results of the avoidance diary should be interpreted with care. Although the avoidance diary was associated with the CAMS at day 30, supporting the diary’s validity, the diary seemed to be a relatively insensitive measure for daily child avoidance. That is, most children reported little to no avoidance over the 30-day period. This may be due to the dichotomous answering option (yes/no avoidance), which could have led children to report only severe instances of avoidance. Third, the 30-day period might not have been long enough to capture avoidance in a community sample. Since a diary is worthwhile to gaining insight into the ecological validity of a questionnaire in a relatively easy and non-invasive manner, developing more extensive diaries (e.g., including daily avoidance rating scales) is an interesting venue, as well as using a diary in a high- or clinically-anxious population where avoidance in daily life is more prevalent. Fourth, although the sample sizes are large enough according to the power analysis, we believe that a larger sample size would be valuable for psychometric studies on assessment tools. Fifth, the community sample was recruited via convenience sampling, whereas the ‘high-anxious’ sample was not, which could result in differences between groups that we are not able to identify or correct.

### Summary

Avoidance is considered a hallmark feature in child anxiety, but convenient measures are scarce. This study examined the psychometric properties of the Child Avoidance Measure (CAM) in a Dutch population, focusing mainly on the child-version. We included children 8 to 13 years old from the community sample as well as a sample of high-anxious children. Regarding the child-version, the internal consistencies were acceptable to good, and its test-retest reliability moderate. The validity analyses showed encouraging results. High-anxious children had higher avoidance scores than children from a community sample. Regarding the parent-version, both the internal consistency and test-retest validity were excellent. Overall, this study supported the sound psychometric properties and usefulness of the CAM. Future studies should focus on the psychometric properties of the Dutch CAM in a clinical sample, assess its ecological validity more extensively, and examine more psychometric features of the parent-version.

## Data Availability

Data will be made available via the Open Science Framework osf.io/2anjd.
